# An Investigation of Employees’ Intention to Comply with Information Security System—A Mixed Approach Based on Regression Analysis and fsQCA

**DOI:** 10.3390/ijerph192316038

**Published:** 2022-11-30

**Authors:** Wenqin Li, Rongmin Liu, Linhui Sun, Zigu Guo, Jie Gao

**Affiliations:** 1 School of Management, Xi’an University of Science and Technology, Xi’an 710054, China; 2 Research Center for Human Factors and Management Ergonomics, Xi’an University of Science and Technology, Xi’an 710054, China; 3 School of Management, Xi’an Jiaotong University, Xi’an 710049, China

**Keywords:** security compliance behavior, PMT, intention to comply, fsQCA, security management

## Abstract

Employee security compliance behavior has become an important safeguard to protect the security of corporate information assets. Focusing on human factors, this paper discusses how to regulate and guide employees’ compliance with information security systems through effective methods. Based on protection motivation theory (PMT), a model of employees’ intention to comply with the information security system was constructed. A questionnaire survey was adopted to obtain 224 valid data points, and SPSS 26.0 was applied to verify the hypotheses underlying the research model. Then, based on the results of a regression analysis, fuzzy set qualitative comparative analysis (fsQCA) was used to explore the conditional configurations that affect employees’ intention to comply with the information security system from a holistic perspective. The empirical results demonstrated that perceived severity, perceived vulnerability, response efficacy, and self-efficacy all positively influenced the employees’ intention to comply with the information security system; while rewards and response costs had a negative effect. Threat appraisal had a greater effect on employees’ intention to comply with the information security system compared to response appraisal. The fsQCA results showed that individual antecedent conditions are not necessary to influence employees’ intention to comply with an information security system. Seven pathways exist that influence an employees’ intention to comply with an information security system, with reward, self-efficacy, and response cost being the core conditions having the highest probability of occurring in each configuration of pathways, and with perceived severity and self-efficacy appearing in the core conditions of configurations with an original coverage greater than 40%. Theoretically, this study discusses the influence of the elements of PMT on employees’ intention to comply with an information security system, reveals the mechanism of influence of the combination of the influencing factors on the outcome variables, and identifies the core factors and auxiliary factors in the condition configurations, providing a new broader perspective for the study of information security compliance behavior and providing some theoretical support for strengthening enterprise security management. Practically, targeted suggestions are proposed based on the research results, to increase the intention of enterprise employees to comply with information security systems, thereby improving the effectiveness of enterprise information security management and the degree of information security in enterprises.

## 1. Introduction

As the information systems of enterprises are highly interactive, shared, and open in the network environment, the application of information technology brings benefits, while also exposing the information systems of enterprises to serious security threats, resulting in many information security incidents. Effective control of unsafe behaviors is an important means of reducing security incidents [1]. Employees’ security behaviors include security compliance behaviors and security engagement behaviors [2]. This paper aimed to investigate employees’ intention regarding security compliance behavior, in the context of information security. Information security risks in enterprises consist of external threats (e.g., virus attacks, cyber espionage, etc.) and internal threats (e.g., inadequate organizational information security management systems, weak information security awareness among employees, etc.). Several studies have shown that internal threats from an organization’s employees have become the main cause of information security incidents [3,4]. In general, the scientific formulation and strict implementation of an information security management system is an effective guarantee of the protection of enterprise information assets [5,6]. On this basis, an increasing number of enterprises have clarified the responsibilities and obligations of employees and formulated a scientific and reasonable information security system. However, in the process of using information assets, employees may invariably ignore the provisions of the information security system, intentionally or unintentionally, thus leading to great threats to information assets such as critical enterprise data and computer equipment [7,8]. Employees are an essential part of enterprise information security management, and increasing their intention to behave in an information security compliant manner helps enterprises to operate safely and effectively in the internet age. Therefore, it is essential to investigate the factors influencing employees’ intention to comply with information security systems.

The factors influencing employees’ intention to act in accordance with information security have been discussed based on protection motivation theory (PMT), whereas few studies have used all the explanatory factors of threat appraisal and response appraisal in PMT. Workman [9] applied four main explanatory factors in his study, namely perceived severity, perceived vulnerability, self-efficacy, and response efficacy; while Lee [10] added the explanatory variable of response cost to the above. Vance et al. [11] conducted a study of information security policy compliance intention by including all six explanatory variables of threat appraisal and response appraisal. Although PMT has been widely used in the study of information security system compliance intention, inconsistent findings have emerged. Ifinedo [12] concluded that perceived susceptibility, response efficacy, and self-efficacy have a positive effect on employees’ information security system compliance intention, while perceived severity has a negative effect, while response cost is insignificant. On the other hand, Vance et al. [11] argued that perceived severity positively affects employees’ willingness to comply with information security systems, while response cost negatively affects such willingness and perceived susceptibility is insignificant. Moreover, a review of the literature revealed that the current studies on the factors influencing individual intention to comply with security behaviors mostly concerned the influence of a single factor [13,14,15], and there are relatively few studies that examined the influence of a combination of multiple factors on individual intention to comply with security behaviors from a holistic perspective.

Given this, this study intended to identify enterprise employees as the research subjects and discuss the effects of various factors in PMT on employees’ intention to comply with information security systems in their daily work situations. Moreover, the antecedents were selected based on the regression results and the fsQCA approach was applied to conduct a conditional configuration analysis of the factors affecting employees’ compliance intention with information security systems in PMT, in order to provide a theoretical basis for the effective prevention of information security incidents in enterprises.

## 2. Theoretical Basis and Research Hypothesis

### 2.1. Overview of Theory

PMT was first introduced by Rogers (1975) in 1975, to explain the process by which an individual’s motivation to protect arises in the face of environmental threats [16]. PMT mainly consists of threat appraisal and response appraisal, where threat appraisal consists of perceived severity, perceived susceptibility, and reward; and response appraisal consists of response efficacy, self-efficacy, and response cost [11]. The theory suggests that when an individual encounters a threat, the individual makes an assessment of the threat and the means available for response, and then chooses to perform or not perform certain protective behaviors.

Anderson C L and Agarwal R argued that the theory of conservation motivation is one of the most powerful explanatory theories for predicting an individual’s intention to engage in conservation action [17]. Previous literature has indicated that PMT is extensively used in the study of information security behavior [18,19,20,21]. Siponen M et al. [18] found that threat appraisal, self-efficacy, and response efficacy had a significant effect on the intention to comply with information security policies, and Tsai H.S. et al. [18] discussed the role of the factors in PMT in the context of information security, using a household computer user as a research subject. Herath T et al. [22] argued that both threat perception and response perception in PMT have an impact on the intention to comply with the information security system. Vance et al. [11] pointed out that perceived susceptibility and reward in threat appraisal and response efficacy, self-efficacy and response cost in response appraisal all have an impact on security policy compliance behavior. Furthermore, Menard P et al. [23] verified that response efficacy is the factor that has the strongest effect on information security behavioral intention. Employees make decisions on whether to comply with information security policies by assessing the potential threat of non-compliance and the usefulness of compliance in reducing information security incidents.

### 2.2. Research Hypotheses

Information security system refers to the information security standards, procedures, terms, and conditions set by the enterprise and that are required to be observed by employees in order to protect the security of information assets. In this paper, the intention of employees to comply with information security system is defined as their willingness to obey and implement the information security system set by their company.

**Figure 1:**
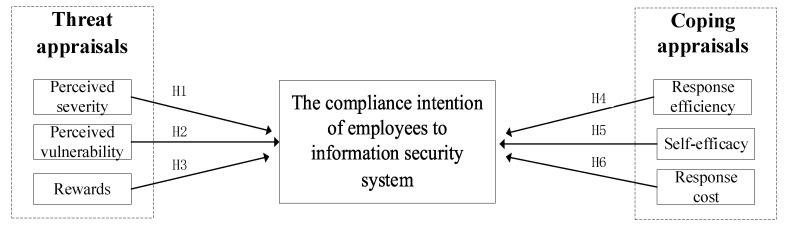
Model of the research hypotheses.

Drawing on previous research models that applied the theory of conservation motivation, based on the expected utility framework, PMT motivates individuals to protect themselves on the basis of a comprehensive threat appraisal and response appraisal [11,12]. An individuals’ intention to perform protective actions is significantly higher if they perceive the severity and vulnerability of the threat, the effectiveness and achievability of the response, and if the cost of the measure is also low [10]. The research model is shown in Figure 1.

Threat appraisal reflects the individual’s assessment of perceived severity, and perceived vulnerability and reward. Perceived severity refers to the severity of the harm caused by a threat, as judged by the individual when confronted with that threat [24]. In this study, perceived severity is defined as the perception by employees of an organization that failure to comply with information security systems will be a security threat that will cause significant damage to the organization. When employees perceive that a security threat will cause significant damage or disruption, they are more likely to feel concerned and will in turn choose to comply with the information security system [22]. Perceived vulnerability refers to the likelihood that an individual perceives how likely he or she is to be harmed [25]. This study defines perceived vulnerability as the likelihood that employees believe that their companies will suffer from information security issues. The more likely an employee perceives that the company will be exposed to a security threat, the more likely they are to adopt protective behaviors. In addition, reward is defined as the benefit of an employee’s non-compliance with an company’s information security system. This study defines rewards as the time, effort, and other benefits that employees save by not complying with the information security system. Since employees often find it easier to carry out their daily work if they do not comply with the company’s information security system, they prefer the option of not complying with the information security system. Therefore, the following hypotheses are proposed:

 **H1.** *Perceived severity has a positive effect on employees’ intention to comply with the information security system.*

 **H2.** *Perceived vulnerability has a positive effect on employees’ intention to comply with the information security system.*

 **H3.** *Reward has a negative effect on employees’ intention to comply with the information security system.*

Furthermore, response appraisal of PMT is an assessment of response efficacy, self-efficacy, and response costs. Response efficacy is the individual’s perception of the action they are taking [26]. If individuals perceive that the measures they undertake are indeed effective, then they will have a higher willingness to adopt the behavior. This study defines response efficacy as the degree to which employees trust that compliance with the information security system is an effective protection of the company’s information systems. The more employees believe that compliance with the information security system can reduce the occurrence of information security incidents, the more likely they will be to comply with the information security system. Self-efficacy refers to individuals’ beliefs regarding their own implementation of information security strategies [27]. In this study, self-efficacy is defined as employees’ beliefs that they can comply with the company’s information security system. As self-efficacy increases, the stronger the individual’s intention to generate protection-motivated behaviors becomes. Response cost is any form of costs associated with information security behaviors and is a countervailing force that prevents people from taking protective action [28]. This study defines reaction costs as the inconvenience of complying with information security systems. Negative feedback that information security management reduces productivity can trigger information security violations by employees [29]. When employees feel that the act of complying with information security systems causes their work to become tedious, their intention to comply with information security systems diminishes. Therefore, the following hypotheses are proposed:

 **H4.** *Reactive efficacy has a positive effect on employees’ intention to comply with the information security system.*

 **H5.** *Self-efficacy has a positive effect on employees’ intention to comply with the information security system.*

 **H6.** *Response cost has a negative effect on employees’ intention to comply with the information security system.*

Employees are motivated toward protective action by an assessment of threat levels and response options. Threat appraisal refers to employees’ awareness that non-compliance with the information security system could make the enterprise more vulnerable to information security incidents and loss of information assets, while response appraisal refers to employees’ belief that they can achieve compliance with the information security system and thus effectively counteract information security threats to the enterprise. Whether the degree of influence of the above two is of equal value in the generation of protection motivation has to be investigated. Therefore, the following hypothesis is proposed:

 **H7.** *Threat appraisal and response appraisal differ in their degree of influence on employees’ intention to comply with the information security system.*

## 3. Regression Analysis

### 3.1. Questionnaire Design and Testing

To ensure that the scale has high reliability and validity, the measurement scales used in this study were derived from existing research. The scale for each element of the PMT was modified from the scales of Bulgurcu et al. [30], Vance et al. [11], and Wurtele et al. [31]. The intention to comply with the information security system was measured using a situational questionnaire, with the specific context referenced from the study by Vance et al. [11] and suitably modified to fit this study. The specific questions on the questionnaire are detailed in Appendix A.

In terms of the control variables, various studies have shown that there are significant gender differences in the findings regarding intention to comply with data protection regulations [32]. In general, the older and more experienced the employees are, the more influence this will also have on their information security behavior. Marriage can enhance employees’ sense of responsibility, while education level has an impact on employees’ perceptions, both of which can affect employees’ information security behavior. Based on the above analysis, gender, age, work experience, marital status, and education level were used as control variables in this study. As state-owned enterprises, private enterprises, and joint venture or wholly foreign-owned enterprises attach different levels of importance to enterprise information security, the information security behaviors of managers and junior employees also vary greatly, and thus the nature of the position and type of enterprise were also categorized as control variables.

After the initial design of the scale was completed, 38 employees were randomly selected to take the test. The factor loadings of the questions were all above the benchmark of 0.50, and the validity test was effective. In addition, based on feedback from the participants, the descriptions of the questions were revised, to improve the clarity and comprehensibility.

A total of 325 questionnaires were collected and 101 invalid questionnaires were excluded, resulting in 224 valid questionnaires, with a valid return rate of 68.92%. The results of the descriptive statistics of the sample for this study are detailed in Table 1. Among the respondents, 48.70% were male and 51.30% were female, with an even gender distribution; the distribution of ages and working years was also reasonable.

### 3.2. Common Method Bias Test

To exclude the effect of common method bias, Harman’s single-factor test was applied in the statistical control section of this study. The test results indicated that the variance explained by the first factor was 31.70%, which is less than the benchmark of 40%, excluding the effect of common method bias.

### 3.3. Reliability and Validity Analysis

The reliability and validity of the scales were tested and the results are detailed in Table 2 and Table 3.

In this study, internal consistency reliability (Cronbach’s α) and composited reliability (CR) were used to evaluate the reliability of the scales. Previous studies identified that a Cronbach’s α and CR value greater than 0.70 indicate good reliability of a questionnaire [33]. From Table 2, it can be seen that most of the evaluation indicators of Cronbach’s α and CR values of the variables are greater than the benchmark value of 0.70, indicating that the reliability of the measurement scale is good.

Studies have identified that when the average variable extraction (AVE) for each construct is greater than 0.5 and the factor loading is greater than 0.6, this indicates that the model has good convergent validity; while when the square root of AVE for each variable is much greater than the correlation coefficient between the variables, this indicates that the model has good discriminant validity [34]. As can be seen from Table 2, the AVE values for all the constructs in this study were greater than 0.50 and the factor loadings were greater than 0.6, indicating that the scale has good convergent validity. Table 3 shows that the square root of AVE for each construct is greater than the correlation coefficient between all the variables, indicating that the scale has good discriminant validity.

### 3.4. Descriptive Statistics

The descriptive statistics of this study are presented in Table 4. It shows that the perceived severity, perceived vulnerability, response efficacy, and self-efficacy were all significantly and positively correlated with employees’ intention to comply with the information security system, while rewards and response costs were all significantly and negatively correlated with compliance with the information security system. The results provided a basis for testing the research hypotheses.

### 3.5. Hypothesis Testing

This study tested the hypotheses through linear regression, and the results are presented in Table 5. Model 1 to 7 all used employee information security system compliance intention as the dependent variable. Model 1 used the control variables as the independent variables, while Model 2 to 7 added a series of variables to test the research hypotheses, including perceived severity, perceived susceptibility, reward, response efficacy, self-efficacy, and response cost.

The results of the tests are as follows: Perceived severity had a significantly positive effect on employees’ intention to comply with the information security system (β = 0.26, *p* < 0.001); Perceived susceptibility had a significantly positive effect on employees’ intention to comply with the information security system (β = 0.34, *p* < 0.001); Reward had a significantly negative effect on employees’ intention to comply with the information security system (β = −0.32, *p* < 0.001); Response efficacy had a significantly positive effect on employees’ intention to comply with the information security system (β = 0.34, *p* < 0.001); Self-efficacy had a significantly positive effect on employees’ intention to comply with the information security system (β = 0.47, *p* < 0.001); Response cost had a significantly negative effect on employees’ intention to comply with the information security system (β = −0.21, *p* < 0.01). From the above results, it can be concluded that H1 to H6 were all validated.

In this study, both threat appraisal and response appraisal were analyzed in the same model, and H7 was tested by comparing the regression coefficients of threat appraisal and response appraisal. The results indicated that β1 = 0.21 and β2 = 0.17 (β1 is the regression coefficient of threat appraisal and β2 is the regression coefficient of response appraisal), with β1 > β2, and thus H7 was verified. This is diametrically opposed to the conclusion of Yan et al. (2020) [35] that users’ willingness to secure information in mobile information services is more affected by response appraisal, possibly because the constructs of threat appraisal and response appraisal in the two studies were different. In the former study, threat appraisal was composed of perceived severity and perceived vulnerability, while the response appraisal was composed of response efficacy and self-efficacy. However, this paper added the construct of reward to threat appraisal and response cost to response appraisal, with both reward and response cost having a significant negative effect on employees’ intention to comply with the information security system, which may be the reason for the inconsistency between the findings of this paper and the former study.

## 4. FsQCA of Factors Influencing Employees’ Intention to Comply with Information Security Systems

### 4.1. The Methodology of FsQCA

FsQCA is a computational approach to the analysis of sets based on the principles of Boolean algebra and set theory, with the underlying philosophy of using truth tables and the idea of logical minimization to generalize the patterns presented in case data [36,37]. Traditional research models of correlation theory are all based on the assumptions of reductionism and focus on the analysis of simple linear relationships between individual antecedents and outcomes, without discussion of complex causal relationships that do not have multiple concurrent causes [38]. Exploring the different combinations of factors in which outcomes occur, namely the “equifinality” of complex causal relationships, is one of the strengths of fsQCA [39]. From reviewing the studies that applied qualitative comparative analysis, it was found that a growing number of management research areas have adopted fsQCA. The method has been widely used in management fields such as business management, energy resources, entrepreneurship research, and urban governance. For example, it has been applied to qualitative comparative analysis, to enrich organizational strategy and configuration theory, to explore the configurational pathways leading to energy price stability, and to investigate the role of different combinations of influencing factors on the success of individual entrepreneurship [40,41,42,43].

### 4.2. FsQCA Model

**Figure 2:**
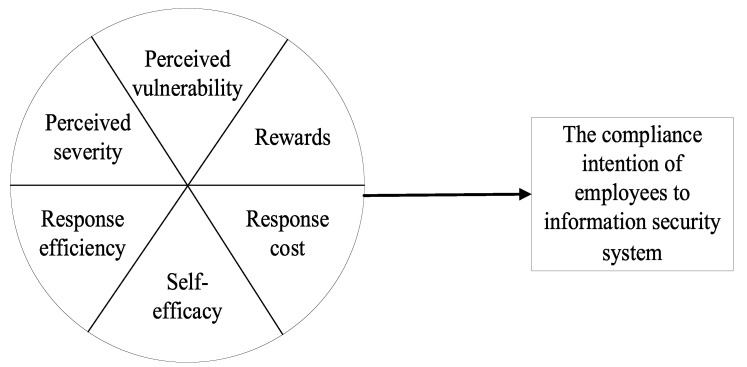
fsQCA model.

The regression analysis results of the elements of PMT revealed that perceived severity, perceived vulnerability, reward, response efficacy, self-efficacy, and response cost are the antecedents that influence employees’ intention to comply with information security systems. However, their compliance intention may not necessarily stem from the influence of a single factor alone, and it is possible for multiple factors to influence the outcome. Although the regression analysis method can analyze the causal relationship between individual antecedent factors and employees’ intention to comply with the information security system, it cannot demonstrate the overall effect of the combination of the antecedent factors. Therefore, this section enriches the findings using fsQCA, to reveal the configuration paths that influenced employees’ intention to comply with the information security system, and tries to explain how different combinations of the above antecedents affected the compliance intention. The fsQCA model is shown in Figure 2.

### 4.3. Data Calibration

To facilitate the operation of the fsQCA software and the simplicity of subsequent descriptions, the antecedent variables involved in this study were labelled with the following abbreviations: PS for perceived severity, PV for perceived vulnerability, R for reward, RE for response efficacy, SE for self-efficacy, RC for response cost, and IB for employees’ intention to comply with the information security system. As the data for this study were obtained from a questionnaire, the sample data were of the numerical type, with a five-point Likert scale. Prior to the data analysis, the sample data needed to be converted into fuzzy set membership scores between 0 and 1: a fuzzy set membership score of 0 means “full non-membership”; fuzzy set membership score of 0.5 is the crossover point between non-membership and membership; and fuzzy set membership score of 1 is “full membership”. The data calibration required the setting of three anchor points: full membership, crossover point, and full non-membership [44]. The percentile was used as an anchor point in the calibration of the Likert scale data [45,46]. Misangyi et al. [47] (2017) stated that specific anchor point percentile values should be determined based on the characteristics of the data distribution. Therefore, this study used the ninety-fifth and fifth quintiles as full membership and full non-membership anchor points for the aforementioned antecedent and outcome variables based on the actual data distribution characteristics, and used the median as the crossover point, which was calculated using the calibrate function in the fsQCA software. Furthermore, the antecedent conditions and anchor values for which calibration had been completed were imported into the software [24]. In terms of analysis, if the case membership value was 0.5, it would be removed due to uncertainty regarding its membership set. To overcome this problem, the calibrated value of 0.5 was converted to 0.501, by increasing by 0.001.

### 4.4. Necessity Analysis

Prior to the qualitative comparative analysis, a necessity analysis was conducted on a single variable, to investigate whether there were necessary conditions for the outcome variable in each of the antecedent variables. In this study, the calibrated values were imported into fsQCA 3.0, and the option “Necessary Conditions” was selected to perform a necessity analysis of the production and non-production of the outcomes, resulting in consistency and raw coverage values for each of the antecedent variables. The consistency value indicates the proportion of antecedent conditions that are necessary for the outcome variable, while the raw coverage value reflects the strength of the explanation of the outcome variable by the antecedent variable. The magnitude of consistency value can determine whether the antecedent variable is necessary or not. When the consistency value of the antecedent variable is higher than 0.9, the variable can be considered necessary for the outcome to occur. It is evident from Table 6 that the consistency values of all the antecedent variables were less than 0.9, so that the explanatory power of a single variable on the outcome variable was insufficient and the configuration effect of the antecedent variables needed to be further explored.

### 4.5. Sufficiency Analysis

The sufficiency analysis included the construction of a truth table and a standardized analysis. The truth table was initially constructed by selecting the truth table production algorithm option “Analyze” in fsQCA 3.0 and incorporating the antecedent and outcome variables into the analysis software, to obtain the truth table. Each row in the truth table represents a possible situation and shows all possible constructs. Second, the consistency and frequency thresholds need to be set, which removes configurations with low consistency or frequency. Fiss [37] (2011) suggested that the consistency threshold can be set to 0.8 for qualitative comparative analysis of fuzzy sets, and the frequency threshold needs to ensure that the number of retained samples is not less than 75% of the number of total samples, which is generally based on the number of samples. The frequency threshold can be set to 1 when the sample size is small, whereas it can be raised as the sample size increases [48,49]. In this study, the consistency threshold was set at 0.8, with the frequency threshold at 2. The existence of contradictory configurations was mitigated by changing the value of the outcome variable of 1 to 0 for PRI values less than 0.7. The data were then standardized, and when selecting the counterfactual analysis, all conditions were selected as “present or absent” in this step, because any of the antecedent variables may have had an impact on employees’ intention to comply with the information security system.

Three types of solutions emerge after the standardized analysis by fsQCA 3.0: complex solutions (conservative solutions that do not incorporate logical remainder terms), simple solutions (incorporating all logical remainder terms), and intermediate solutions (incorporating partially factored logical remainder terms). The results are discussed mainly in terms of intermediate solutions, aided by simple solutions, to identify the core and auxiliary conditions [37]. The analysis of the intermediate and simple solutions yielded seven configurations that produced results for employees’ intention to comply with the information security system (see Table 7).

From the above results, it can be concluded that seven different condition configurations leading to the result that employees’ intention to comply with the information security system were generated. The preliminary analyses of the seven conditional configurations are as follows:

Configuration 1a: PS*PV*~R*SE. This indicates that employees with a high perceived severity, perceived vulnerability, and self-efficacy but low rewards have a stronger intention to comply with the information security system, regardless of their response effectiveness and response cost. In this configuration, high perceived severity, high self-efficacy, and low rewards are the core conditions.

Configuration 1b: PS*~R*RE*SE. This indicates that employees with a high perceived severity, response efficacy, and self-efficacy but low rewards have a stronger intention to comply with the information security system, regardless of their perceived vulnerability and response cost. In this configuration, high perceived severity, high self-efficacy, and low rewards are the core conditions.

Configuration 2a: ~PV*~R*RE*SE*~RC. This indicates that employees with a high response efficacy and self-efficacy but low perceived vulnerability, rewards, and response costs have a stronger intention to comply with the information security system, regardless of their perceived severity. In this configuration, high response efficacy, low rewards, and low response costs are the core conditions.

Configuration 2b: PS*PV*~R*RE*~RC. This indicates that employees with a high perceived severity, perceived vulnerability, and response efficacy, but low reward and response cost have a stronger intention to comply with the information security system, regardless of their self-efficacy. In this configuration, high response efficacy, low rewards, and low response costs are the core conditions.

Configuration 3: PS*~R*SE*~RC. This indicates that employees with a high perceived severity and high self-efficacy but low reward and response costs have a stronger intention to comply with the information security system, regardless of their perceived vulnerability and response efficacy. In this configuration, high perceived severity, high self-efficacy, low rewards, and low response costs are the core conditions.

Configuration 4: PS*RE*SE*~RC. This indicates that employees with a high perceived severity, response efficacy, and self-efficacy, but low response cost, have a stronger intention to comply with the information security system, regardless of their perceived vulnerability and rewards. In this configuration, high perceived severity, high self-efficacy, and low response cost are the core conditions.

Configuration 5: PV*R*RE*SE*~RC. This indicates that employees with a high perceived vulnerability, rewards, response efficacy, and self-efficacy, but low response costs, have a stronger intention to comply with the information security system, regardless of their perceived severity. In this configuration, high perceived susceptibility, high response efficacy, high self-efficacy, and low response cost are the core conditions.

## 5. Result and Discussion

### 5.1. Discussion of the Regression Analysis Results

Focusing on employees in domestic enterprises, this paper confirms that all elements of PMT have significant effects on employees’ intention to comply with the information security system: perceived severity, perceived vulnerability, response effectiveness, and self-efficacy all have positive effects on employees’ intention to comply with the information security system, the finding regarding perceived severity is consistent with the finding of Vance et al. [11], and perceived susceptibility, response efficacy, and self-efficacy were validated, with the same findings as in Ifinedo’s study [12]; while rewards and response costs have negative effects on compliance intention, which is consistent with the findings of Vance et al. [11]. This suggests that employees are more likely to make the decision to comply with the information security system if the perceived security threat from non-compliance is greater and the ease of perceiving the threat is higher, and if they believe that compliance with the information security system can effectively counteract the information security threat and perceive a high likelihood that they can achieve this. On the other hand, employees are more likely to choose not to comply with information security systems if they perceive that the benefits are greater and the costs of compliance are higher. The basic assumption of the theory of rational behavior is that people are rational and consider the implications and consequences of their actions based various information before making a decision. The results of this study are consistent with the basic assumptions of the theory of rational behavior, in that both the threat appraisal (perceived severity, perceived vulnerability, and reward) and response appraisal (response efficacy, self-efficacy, and response cost) have an impact on employees’ intention to comply with the information security system in their own behavioral intention.

To the extent that it influences employees’ intention to comply with information security systems, threat appraisal plays a greater role than response appraisal. The loss aversion effect in psychology states that the negative utility from an equivalent loss is 2.5 times greater than the positive utility from an equivalent gain. Threat appraisal refers to the assessment of the threat posed by an employee’s non-compliance with the information security system and provides negative utility to the employees. In contrast, response appraisal refers to the assessment of the likelihood of employees complying with the information security system, in order to respond to information security threats, and provides positive utility to employees. Hence, the utility value of threat appraisal is greater than that of response appraisal. According to the utility theory, it can be concluded that the assessment of external information security threats is more influential in employees’ intention to comply with an information security system than their assessment of whether they have the ability to respond to and avoid threats.

### 5.2. Discussion of the fsQCA Results

Three values are present for all the configurations in the fsQCA analysis results: consistency, raw coverage, and unique coverage. Specifically, consistency is the probability that a certain combination of antecedent variables would produce the outcome variable; raw coverage is the proportion of the case configuration across all the cases produced by the results; and unique coverage is the proportion of the cases produced by the results that can only be explained by that configuration. From Table 7, it can be seen that the consistency of configurations 1–5, as well as the solution, is higher than the theoretical value (0.8), suggesting that the reliability of the above configurations was superior and that all of the above seven configurations were sufficient conditions to lead to the generation of employees’ intention to comply with the information security system. The raw coverage of each conditional configuration was higher than 20%, indicating that each configuration can explain more than 20% of the total sample. Moreover, the overall coverage of the solution was 0.630714, covering approximately 63% of the case sample, indicating that the above seven conditional configurations can explain more than 63% of the cases of employee information security system compliance intention generation in the total sample, among which conditional configuration 1a had the highest raw coverage and a stronger explanation for the generation of the outcome variables.

Comparison of the seven configurations reveals that: (1) Reward, self-efficacy, and response cost appear as core conditions in five of the configurations with the highest probability of occurrence. This indicates that rewards, self-efficacy, and response costs are important conditions for increasing employees’ intention to comply with the information security system. Therefore, in order to increase employee’s compliance intentions, it is necessary to start by improving employees’ self-efficacy and reducing the rewards associated with perceived non-compliance and the costs resulting from their compliance behavior. (2) Response cost is a core condition whenever it appears in a configuration, and it appears in all five configurations. This suggests that response cost plays a pivotal role in promoting employees’ intention to comply with the information security system relative to other variables. (3) A comparative analysis of the raw coverage of each configuration showed that perceived severity and self-efficacy appear in the core conditions of all the configurations with a raw coverage greater than 40%. This indicates that enhancing employees’ perceived severity and self-efficacy can maximize employees’ intention to comply with the information security system within an organization.

## 6. Conclusions and Prospects

### 6.1. Reflections on Management

In light of the regression analysis results, enterprises should regularly conduct information security education and training for employees. The ability of employees to perceive information security threats can be enhanced by learning about information security cases in the same industry, and the perceived severity and perceived vulnerability of employees can be improved, thus increasing their willingness to comply with the information security system and contributing to the sustainable development of the enterprise. Moreover, it is recommended that lectures and communication sessions related to enterprise information security be held regularly. Through information security lectures and communication sessions, employees can experience a positive information security atmosphere in the enterprise, which may facilitate employees’ effectiveness of response and self-efficacy. This would promote the positive behavior of employees in complying with the information security system, effectively protecting enterprise information assets and improving the level of enterprise security management.

Based on the fsQCA results, it is evident that increasing employees’ perceived severity and self-efficacy, as well as reducing employees’ perceived rewards for security non-compliance, would have the strongest effect on improving employees’ intention to comply with information security systems, considering the raw coverage of each configuration effect. Companies could design targeted training courses that focus on improving employees’ perceived severity and self-efficacy. In addition, information security system regulations could be optimized, with reference to multiple opinions, improving the scientific basis of information security systems, to reduce employees’ perceptions of rewards for security non-compliance.

### 6.2. Limitations and Prospects

There are some limitations in this study. First of all, when measuring the dependent variable of employees’ intention to comply with the information security system, a scenario-based questionnaire was adopted, and the specific scenarios in the questionnaire were designed with reference to existing studies and in line with actual situations, but other information security behavior scenarios were not taken into account. In further research, more scenarios could be selected through observation by conducting in-depth field research. Moreover, this study merely considered individual influences, while organizational contextual factors can also influence individual behavior, and future studies may consider adding organizational factors.

## Figures and Tables

**Table 1:** Participants’ characteristics.

Measure	Items	Frequency	Percent
Gender	Male	109	48.70
	Female	115	51.30
Age	30 years and below	107	47.80
	31 to 40 years	44	19.60
	41 to 50 years	58	25.90
	Above 50 years	15	6.70
Marital status	Married	123	54.90
	Unmarried	101	45.10
Education	Below college	18	8.00
	College and undergraduate	145	64.70
	Graduate	61	27.20
Work experience	5 years or less	101	45.10
	6 years to 10 years	23	10.30
	11 years to 15 years	26	11.60
	16 years or more	74	33.00
Work nature	Grass-roots staff	151	67.40
	Middle-level grass-roots managers	60	26.80
	Senior managers	13	5.80
Enterprise type	Software and information services industry	65	29.00
	Not in the software and information services industry	159	71.00

**Table 2:** Measurement scales and evaluation indicators for reliability and validity.

Variable	Term	Factor Loading	AVE	CR	Cronbach’s α
Perceived severity	PS1	0.84	0.76	0.91	0.84
	PS2	0.89			
	PS3	0.89			
Perceived vulnerability	PV1	0.63	0.67	0.86	0.74
	PV2	0.92			
	PV3	0.88			
Reward	R1	0.81	0.67	0.86	0.75
	R2	0.89			
	R3	0.74			
Response efficacy	RE1	0.82	0.70	0.87	0.78
	RE2	0.87			
	RE3	0.82			
Self-efficacy	SE1	0.88	0.77	0.87	0.70
	SE2	0.88			
Response cost	RC1	0.78	0.52	0.81	0.69
	RC2	0.75			
	RC3	0.72			
	RC4	0.62			
The compliance intention of employees with the information security system	IB1	0.81	0.69	0.93	0.91
	IB2	0.86			
	IB3	0.87			
	IB4	0.87			
	IB5	0.73			
	IB6	0.83			

**Table 3:** Discriminant validity test results.

Variable	Perceived Severity	Perceived Vulnerability	Reward	Response Efficacy	Self-Efficacy	Response Cost	The Compliance Intention of Employees with the Information Security System
Perceived severity	**0.87**	-	-	-	-	-	-
Perceived vulnerability	0.57	**0.82**	-	-	-	-	-
Reward	−0.38	−0.24	**0.82**	-	-	-	-
Response efficacy	0.46	0.56	−0.31	**0.83**	-	-	-
Self-efficacy	0.26	0.29	−0.33	0.33	**0.88**	-	-
Response cost	−0.05	0.04	0.32	−0.06	−0.27	**0.72**	-
The compliance intention of employees to information security system	0.33	0.36	−0.43	0.38	0.47	−0.21	**0.83**

Note: Diagonal black bold numbers are the square root of AVE values; correlation coefficients are in the lower triangle of the matrix.

**Table 4:** Mean, standard deviation, and correlation coefficient.

Variable	Mean Value	Standard Deviation	Perceived Severity	Perceived Vulnerability	Reward	Response Efficacy	Self-Efficacy	Response Cost
Perceived severity	3.97	1.01	-	-	-	-	-	-
Perceived vulnerability	3.98	0.90	-	-	-	-	-	-
Reward	2.24	1.04	-	-	-	-	-	-
Response efficacy	4.05	0.91	-	-	-	-	-	-
Self-efficacy	4.17	0.82	-	-	-	-	-	-
Response cost	2.86	0.89	-	-	-	-	-	-
The compliance intention of employees with the information security system	4.40	0.81	0.33 ***	0.36 ***	−0.43 ***	0.38 ***	0.47 ***	−0.21 **

Note: ** is *p* < 0.01, *** is *p* < 0.001.

**Table 5:** Results of the regression analysis.

Variable		Dependent Variable: The Compliance Intention of Employees with Information Security System						
		Model 1	Model 2	Model 3	Model 4	Model 5	Model 6	Model 7
Independent variables	Perceived severity	-	0.26 ***	-	-	-	-	-
	Perceived vulnerability	-	-	0.34 ***	-	-	-	-
	Reward	-	-	-	−0.32 ***	-	-	-
	Response efficacy	-	-	-	-	0.34 ***	-	-
	Self-efficacy	-	-	-	-	-	0.47 ***	-
	Response cost	-	-	-	-	-	-	−0.21 **
Control variables	Gender	0.35 **	0.31 **	0.32 **	0.30 **	0.33 **	0.34 **	0.31 **
	Age	0.01	0.04	0.04	0.02	0.08	0.08	0.00
	Marital status	−0.09	−0.03	−0.15	−0.06	−0.15	−0.12	−0.08
	Education	−0.01	−0.05	−0.10	0.03	−0.01	0.03	0.06
	Work experience	−0.09	−0.12	−0.16	−0.08	−0.17	−0.17	−0.10
	Work nature	0.21	0.25 *	0.19	0.18	0.16	0.16	0.26 *
	Enterprise type	0.01	−0.01	−0.06	−0.04	−0.05	−0.06	0.01
R2		0.06	0.16	0.19	0.22	0.20	0.28	0.11
ΔR2		0.03	0.13	0.16	0.19	0.17	0.25	0.07
*F*-value		1.90	5.21 ***	6.34 ***	7.63 ***	6.70 ***	10.33 ***	3.19 **

Note: * is *p* < 0.05, ** is *p* < 0.01, *** is *p* < 0.001.

**Table 6:** Analysis of necessary conditions.

Antecedent Variables	IB		~IB	
	Consistency	Coverage	Consistency	Coverage
PS	0.743281	0.733336	0.554426	0.415710
~PS	0.407787	0.546332	0.644351	0.656058
PV	0.732438	0.751328	0.545576	0.425315
~PV	0.439765	0.560130	0.681012	0.659204
R	0.456366	0.525043	0.768704	0.672105
~R	0.714996	0.802669	0.456779	0.389704
RE	0.729657	0.733976	0.552038	0.422016
~RE	0.425418	0.555481	0.652012	0.647002
SE	0.744805	0.777454	0.505711	0.401172
~SE	0.426321	0.531596	0.719461	0.681786
RC	0.531048	0.604191	0.678882	0.586991
~RC	0.636992	0.723008	0.542227	0.467721

Note: The “~” denotes the logical relationship “not”.

**Table 7:** Configuration results of employees’ intention to comply with the information security system.

Conditional Configuration	The Compliance Intention of Employees to Information Security System						
	1a	1b	2a	2b	3	4	5
PS	●	●		●	●	●	
PV	●		⊗	●			●
R	⊗	⊗	⊗	⊗	⊗		●
RE		●	●	●		●	●
SE	●	●	●		●	●	●
RC			⊗	⊗	⊗	⊗	⊗
Consistency	0.898646	0.874394	0.892613	0.907320	0.900102	0.888571	0.878373
Raw coverage	0.468906	0.464805	0.227075	0.382746	0.412367	0.402617	0.201320
Unique coverage	0.018480	0.015392	0.012877	0.040809	0.013035	0.011165	0.009821
Consistency between solutions	0.855480						
Coverage between solutions	0.630714						

Note: “●” signifies that the condition exists, and “⊗” signifies that the condition does not exist. A larger circle represents the condition as a core condition, while a smaller circle represents the condition as a secondary condition. Blanks show that the presence or absence of the condition did not affect the results.

## Data Availability

Most of the data of this study are presented, and the remainder can be obtained from the corresponding author upon reasonable request.

## Appendix A

This section gives the questionnaire applied to obtain the research data needed for this study.

Questionnaire for the study of insecure behavior in the context of information security:

Hello! First of all, thank you very much for participating in this survey during your busy schedule. This is a purely academic and anonymous research questionnaire, which aims to study the insecure behavior of employees in the context of information technology and its influencing factors. The questionnaire is anonymous and the survey does not involve business secrets, and there are no right or wrong answers. Your support is crucial to our research. Once again, we express our sincere gratitude for your cooperation!

Questionnaire instructions: 1. There is only one answer for each question; 2. Fill in the questionnaire according to your actual situation; 3. Please try to answer each question and do not miss anything. Please tick on the options.

**Part I**

Gender: (1) Male (2) Female

Age: (1) 30 years and below (2) 31 to 40 years (3) 41 to 50 years (4) above 50 years

Marital status: (1) married (2) unmarried

Education: (1) below college (2) college and undergraduate (3) graduate

Working years: (1) 5 years or less (2) 6 years to 10 years (3) 11 years to 15 years (4) 16 years or more

Job nature: (1) grass-roots staff (2) middle-level grass-roots managers (3) senior managers

Enterprise type: (1) state-owned enterprises (2) private enterprises (3) joint ventures or wholly-owned foreign enterprises

Whether the company belongs to the software and information technology service industry:

(1) Yes (2) No

**Part II**

The following questions reflect some situations at work, so please choose according to your true feelings. For each question, choose from the 5 levels of the scale below, and tick the level that best fits your situation, without duplication.

[1: “Completely disagree (not at all consistent)”, 2: “Less agree (less consistent)”, 3: “Not sure”, 4: “More agree (more consistent)”, 5: “Completely agree (fully consistent)”]

**Serial No.**	**Title Items**	**Likert Scale of 5 Levels**				
		**1**	**2**	**3**	**4**	**5**
1	If I copy some of my company’s confidential data to unencrypted portable media (such as a USB drive), it can create serious information security problems for my company.					
2	If I do not comply with the information security system, the company I work for will face serious information security problems.					
3	For me, the loss of data privacy by not complying with the information security system is a serious issue.					
4	The work computer was invaded by a virus and the company’s information security system requires contacting a professional to remove the virus. If I choose to solve the virus problem myself, I can save my work time.					
5	The company’s information security system prohibits copying this data to unencrypted portable media (e.g., USB drives), and it saves me time at work if I copy the data to a USB drive to facilitate analysis of the data during a business trip.					
6	Failure to comply with the information security system saves time on the job.					
7	If I do not comply with the information security system, I may be exposed to information security threats.					
8	If I do not follow the information security system, the company I work for may have information security problems.					
9	The company’s information security system states that passwords are not to be shared, and if I share my password with a colleague, there may be information security issues.					
10	Compliance with the information security system can reduce the probability of information system security problems.					
11	Comply with the information security system, and there will be few information system security problems.					
12	Compliance with the information security system can prevent security breaches in the company’s information system.					
13	It is easy for me to comply with the information security system established by the company.					
14	I am able to consciously comply with the information security system.					
15	Compliance with the information security system inconveniences my daily work.					
16	Compliance with the information security system incurs associated administrative costs.					
17	Complying with the information security system not only takes time but also requires a lot of effort.					
18	Compliance with information security systems can be a waste of work time.					
The following six items are situational questions. In the following scenarios, choose whether you agree with what the people in the scenario are doing on the 5-point scale below.						
19	Ming walks to the shared office printer alone and sees a document printed by someone else. The document is marked as “confidential”. The information security system prohibits reading confidential information, but Ming is curious and chooses to read the document. Do you agree with Ming’s action?					
20	Jun is browsing a website that may be problematic at work when an anti-virus program alerts him that his computer has been invaded by a virus. Although the information security system requires contacting a professional to remove the virus, Jun chooses to solve the virus problem himself for convenience. Do you agree with Jun’s approach?					
21	Li takes her work laptop home to work. Her children want to use the laptop to play games. Although her company’s information security system prohibits sharing the work computer with anyone. However, Li gives the laptop to her children to use. Do you agree with what Li is doing?					
22	Le is working in a position that requires him to know the personnel details of the company. His company’s information security system prohibits copying this data to unencrypted portable media (such as a USB drive). However, Le is on a business trip and he wants to analyze the personnel data during the trip, so he chooses to copy this data to his own portable USB drive. Do you agree with Le’s approach?					
23	The information security system at Hong’s company requires all users to lock their computers every time they leave them. Hong’s supervisor asks Xiaohong to unlock her computer before she leaves so that other employees can use it. So Hong unlocks the computer before she leaves. Do you agree with Hong’s approach?					
24	Kai uses a file server at work, which he can access by entering a password. His company’s information security system states that passwords are not to be shared. Kai is on a business trip and one of his colleagues needs the files on the file server. So Kai tells his colleague the password. Do you agree with Kai’s approach?					
